# The results of bone deformity correction using a spider frame with web-based software for lower extremity long bone deformities

**DOI:** 10.1051/sicotj/2016005

**Published:** 2016-03-22

**Authors:** Ali Çağrı Tekin, Haluk Çabuk, Süleyman Semih Dedeoğlu, Mehmet Selçuk Saygılı, Müjdat Adaş, Cem Zeki Esenyel, Cem Dinçay Büyükkurt, Murat Tonbul

**Affiliations:** 1 Department of Orthopaedics and Traumatology, Okmeydanı Training and Research Hospital 34384 Istanbul Turkey; 2 Metin Sabanci Baltalimani Bone Disease Training and Research Hospital 34470 Istanbul Turkey; 3 Privite Reyap Hospital Tekirdağ Turkey

**Keywords:** Computer-assisted software, Deformity correction, Limb shortness treatment

## Abstract

*Aim*: To present the functional and radiological results and evaluate the effectiveness of a computer-assisted external fixator (spider frame) in patients with lower extremity shortness and deformity.

*Materials and methods*: The study comprised 17 patients (14 male, 3 female) who were treated for lower extremity long bone deformity and shortness between 2012 and 2015 using a spider frame. The procedure’s level of difficulty was determined preoperatively using the Paley Scale. Postoperatively, the results for the patients who underwent tibial operations were evaluated using the Paley criteria modified by ASAMI, and the results for the patients who underwent femoral operations were evaluated according to the Paley scoring system. The evaluations were made by calculating the External Fixator and Distraction indexes.

*Results*: The mean age of the patients was 24.58 years (range, 5–51 years). The spider frame was applied to the femur in 10 patients and to the tibia in seven. The mean follow-up period was 15 months (range, 6–31 months) from the operation day, and the mean amount of lengthening was 3.0 cm (range, 1–6 cm). The mean duration of fixator application was 202.7 days (range, 104–300 days). The mean External Fixator Index was 98 days/cm (range, 42–265 days/cm). The mean Distraction Index was 10.49 days/cm (range, 10–14 days/cm).

*Conclusion*: The computer-assisted external fixator system (spider frame) achieves single-stage correction in cases of both deformity and shortness. The system can be applied easily, and because of its high-tech software, it offers the possibility of postoperative treatment of the deformity.

## Introduction

One of the greatest problems with correcting a complex deformity with an Ilizarov circular external fixator is the need to modify to the system to prevent residual deformity.

If the deformity is to be treated gradually, then angulation, lengthening, rotation, and translation treatments must be performed sequentially [1]. In cases of complex deformities treated with an Ilizarov circular external fixator that require the modification and prolongation of the correction period, external fixator systems with various types of computer assistance have been used for the last 10 years [2].

In this study, the Spider Frame^®^ computer-assisted circular external fixator system was selected as a hexapod external fixator for use with patients with deformities. The aim of this study was to evaluate the results and effectiveness of a computer-assisted external fixator (Spider Frame) and high-tech software (Spider Frame Correction Software) used to treat shortness and deformity.

## Patients and methods

After the protocol was approved by our institutional review boards, we performed a retrospective study. A total of 17 patients (14 male, 3 female) with lower limb-length discrepancy and deformity were treated using a computer-assisted external fixator (Spider Frame). The mean age of the patients was 24.58 years (range, 5–51 years). The deformities were in the femur in 10 cases and in the tibia in seven.

The causes of the femur deformities were malunion after trauma in five cases (50%), congenital deformity in four cases (40%), and Perthes disease sequelae in one case (10%). The causes of the tibia deformities were congenital deformity in three cases (42.8%), malunion in two cases (28.5%), osteogenesis imperfecta in one case (14.2%), and multiple endochondromatosis in one case (14.2%). The deformity was in the oblique plane in five cases and in the coronal plane in six cases; in one case, it was an isolated rotational deformity. In five cases, there was isolated shortness. Direct radiographs were used in the radiological evaluation of all patients. Additional computed tomography (CT) imaging was used for only one patient, in whom rotational deformity was suspected. The deformities were analyzed, and the radiological results were evaluated.

During the preoperative evaluation, the Paley Scale of Difficulty was used. According to this classification, the cases were graded as mild (0–6 points), moderate (7–11 points), and difficult (>12 points) [3]. The Paley scoring system was used again for femoral lengthening [3]. According to this scoring system, which was based on radiological and clinical parameters, the scores were evaluated as excellent (95–100), good (75–94), fair (40–74), and poor (<40). The results of the tibial deformity treatment were evaluated using the Paley criteria modified by the Association for the Study and Application of Methods of Ilizarov (ASAMI) criteria as excellent, good, fair, and poor [4]. In the ASAMI criteria, excellent scores indicate union, no infection, deformity under 7°, and limb-length discrepancy under 2.5 cm; good scores indicate union and two of the above criteria; fair scores indicate union and only one of the above criteria; and poor scores indicate non-union, refracture, union and infection, deformity greater than 7°, or limb-length discrepancy over 2.5 cm [4].

For all patients, the Distraction Index and the External Fixator Index were calculated. The Distraction Index value was the total length obtained divided by the number of days of the distraction period. The External Fixator Index value was obtained by dividing the total duration of fixator application by the total length gained.

## Surgical technique

Under general anesthesia and after sterile draping of the extremity, the joint lines and the osteotomy line were identified from the CORA points of the measurements that were analyzed. The rings close to the joint area were then fixed with at least three Schanz screws to each ring in the femur and with a maximum of three Schanz screws and 1 K-wire in the tibia, ensuring that the screws were parallel to the joint and perpendicular to the mechanical axis of the bone. All of the Schanz screws were 6-mm-diameter stainless steel with hydroxyapatite coating. When preparing the Spider Frame, 2/3 rings around the knee were preferred to avoid restricting joint movement. After the fixation of the rings perpendicular to the bone segments, a total of six struts were placed between the two rings to connect them. After the numbers on the struts were entered into the computer program, the osteotomy was performed with percutaneous multidrilling in the femur and with a percutaneous Gigli saw from the metaphysio-diaphyseal area in the tibia. After visualization of the complete fracture and separation under fluoroscopy, the two rings were connected by bringing them to the distance of the previously recorded strut length. The previous image of the deformity was confirmed with another fluoroscopy image. After the wound was closed, an aluminum sphere with specific measurements was fixed to the reference ring. The fibular osteotomy was also performed, ensuring that it was not at the same level as the tibial osteotomy.

**Figure 1.**
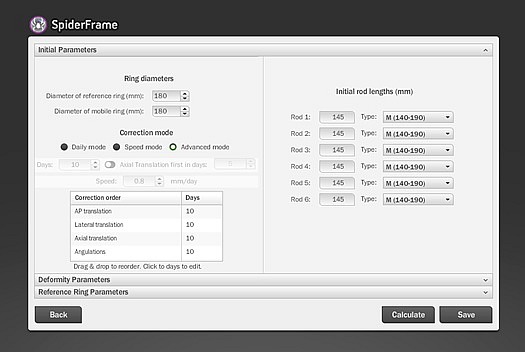
Spider Frame correction modes.

**Figure 2.**
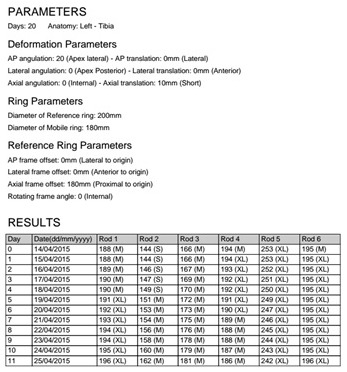
An example of a correction prescription.

In all patients, postoperative anterior-posterior (AP) and lateral radiographs of the extremity were taken on the same day. The ring dimensions, angular defects, and amounts of shortness were calculated on the radiographs, and prescriptions for correction were created by entering the data into the web-based software (Figure 1). The lengthening and deformity correction procedures usually began on the 10th postoperative day (range, 7–14 days). One week after the postoperative correction began, radiographs were taken, and the accuracy of the correction was determined using both the radiograph and the prescription created with the software (Figure 2). When the prescribed protocol was finished, follow-up orthoroentgenograms were taken of all the patients; if there was residual deformity, a corrective prescription was created with the software.

**Figure 3.**
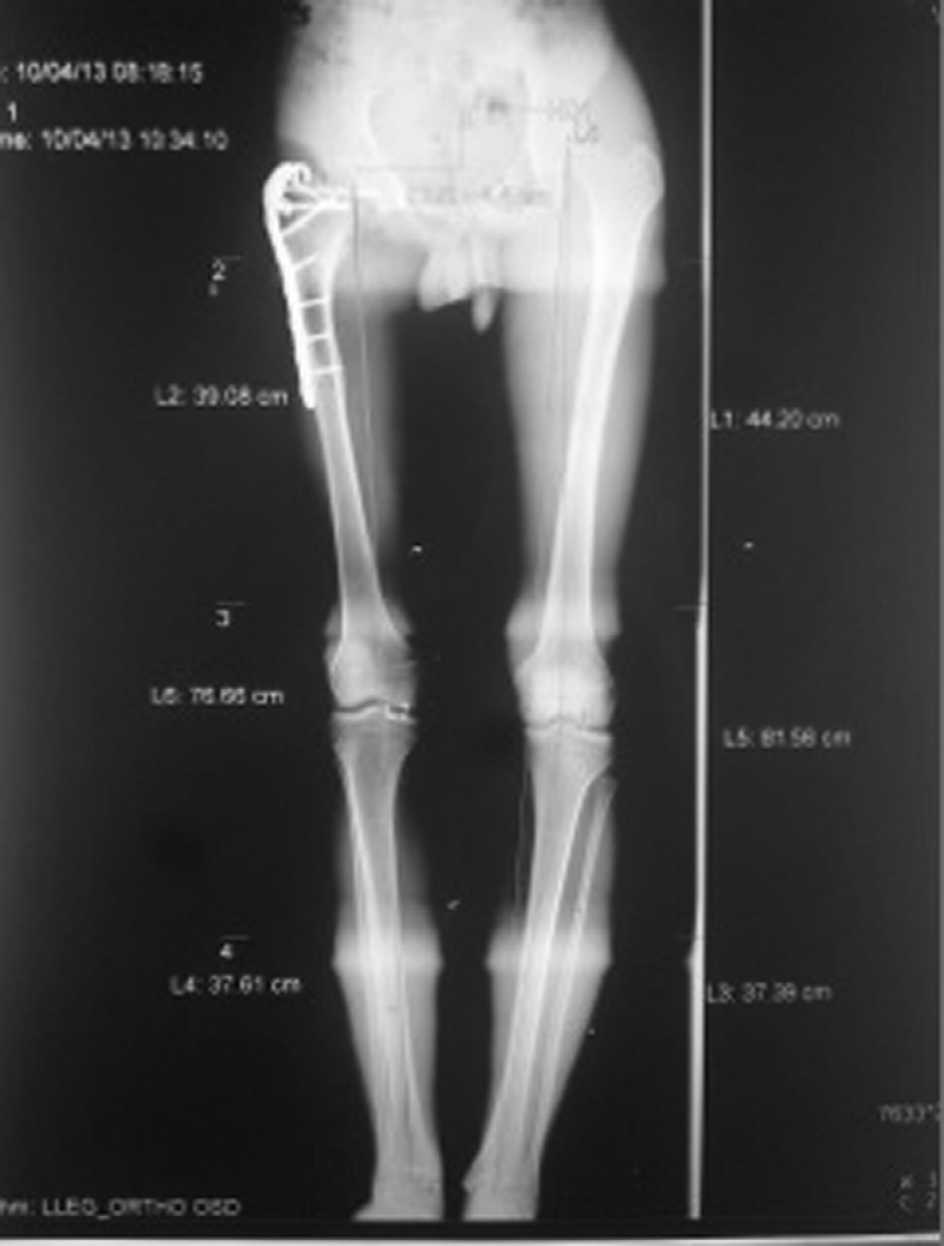
A 17-year-old male with Perthes disease sequelae and preoperative 4-cm shortness of femur (Case No. 5 in Table 1).

**Figure 4.**
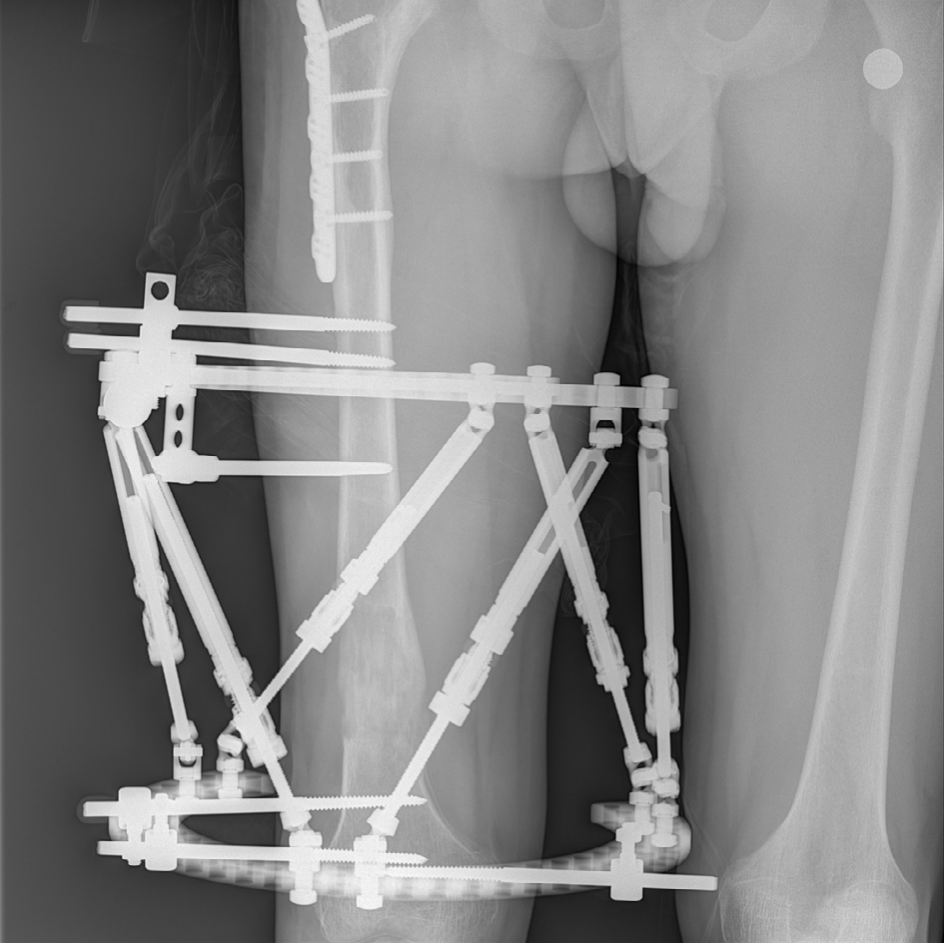
Same patient after correction with the Spider Frame (Case No. 5 in Table 1).

**Figure 5.**
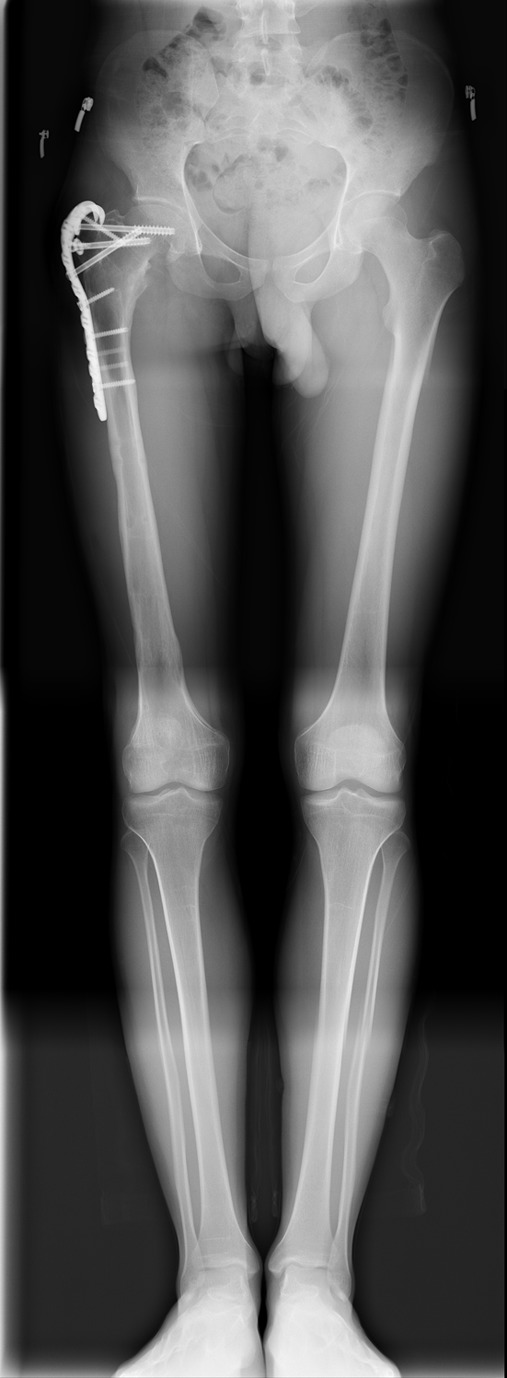
Same patient after the removal of external fixation (Case No. 5 in Table 1).

**Figure 6.**
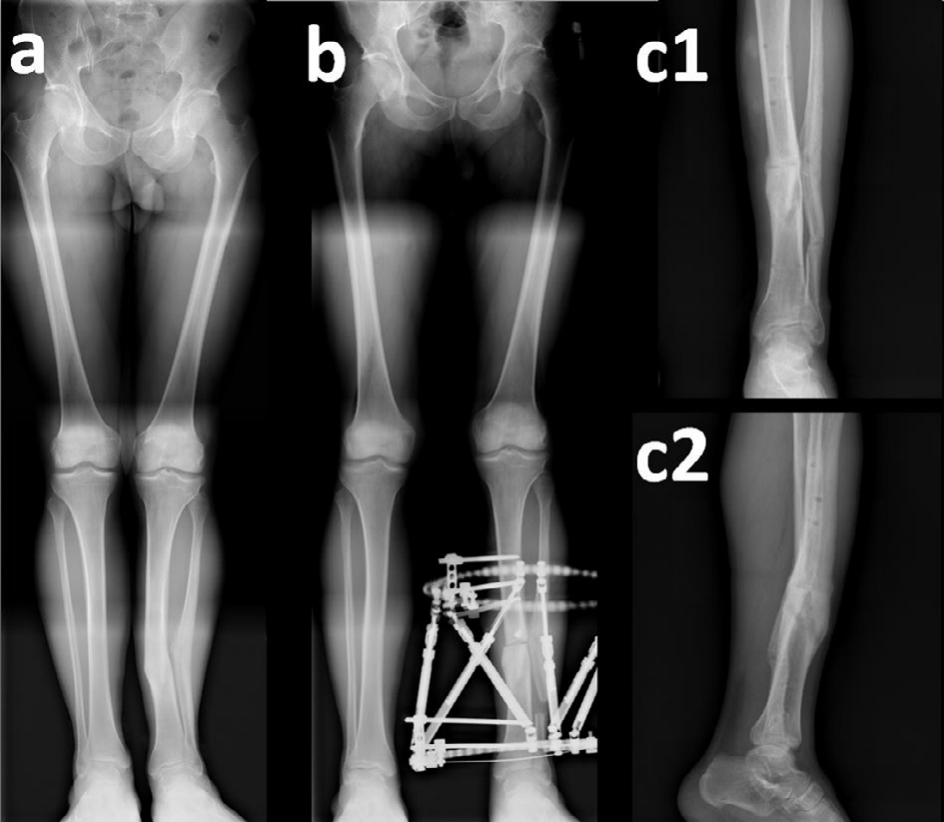
Case No. 17 in Table 1. (a) Valgus, procurvatum and rotation deformity of tibia, (b) after correction with Spider Frame, (c1) and (c2) anteroposterior and lateral X-rays of same patient after removal of Spider Frame.

**Figure 7.**
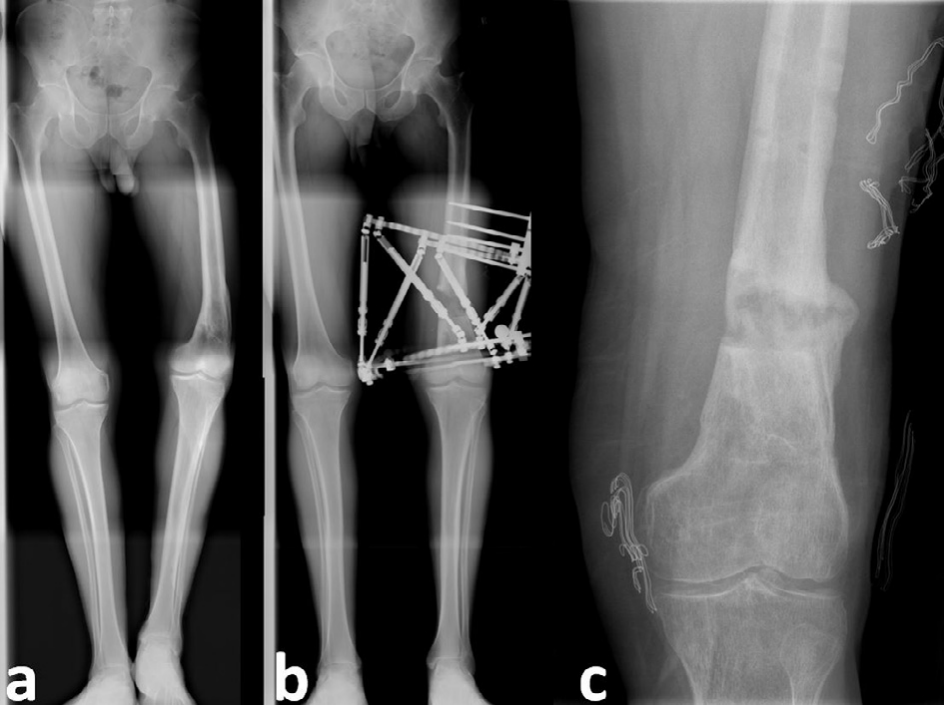
Case No. 6 in Table 1. (a) Varus deformity of femur with preoperative 5-cm shortness, (b) same patient after correction with the Spider Frame, (c) same patient after the removal of Spider Frame.

The patients were given detailed information and shown how the prescriptions would be applied using the system. The struts were changed in the orthopedic clinic under the observation of the doctor. The external fixator was applied for 30 days for each cm of lengthening needed, and follow-up continued until callus tissue could be visualized on three sides on the direct radiograph. When full union was achieved and callus tissue was observed on three sides, the fixator system was removed (Figures 3–7).

## Results

The mean follow-up period was 15.4 months (range, 6–31 months). The mean duration of external fixator application was 202.7 days (range, 104–300 days), including 196.4 days (range, 104–266 days) in the femur cases and 211.7 days (range, 114–300 days) in the tibia cases. The mean lengthening of the bone was 3.0 cm (range 1–6 cm). The lengthening of the bone was <3 cm in eight patients and ≥3 cm in nine patients.

The mean period of gradual correction to eradicate the angular deformity and shortness was 30 days (range, 10–60 days): 35.2 days in femur cases and 22.4 days in tibia cases. The mean Distraction Index was 10.14 days/cm in the femur cases and 11.0 days/cm in the tibia cases. The mean External Fixator Index was 98 days/cm (range, 42–265 days/cm).

The deformities were in the oblique plane in five cases and the coronal plane in six cases. There was isolated shortness in five cases and isolated rotation deformity in one case. The mean angular deformity was 18° (range, 8°–30°). The deformities in the 17 bone segments were all corrected to the ideal form.

The preoperative Paley Scale of Difficulty score [5] was 3.82 (range, 1–7). According to this scale, 16 cases were of mild difficulty and one was of moderate difficulty. No cases scored 12 points or higher, which would indicate severe difficulty.

At the end of the follow-up period, the Paley scoring system was used for the patients who had undergone femoral lengthening. The mean score was 87 (range, 65–95). The cases in the current study were evaluated, with four showing excellent results, five showing good results, and one showing fair results.

In the evaluation following tibial correction, the Paley criteria modified by ASAMI were used. These criteria indicated that functional and bone results were obtained. The bone results were evaluated as excellent in five cases, good in one case, and poor in one case. The functional results were excellent in four cases and good in three cases (Table 1).

**Table 1.** Master table of all patients.

No	Sex	Etiology	Deformity	App period of ex-fix	Correction period	Lengthening (cm)	Distraction index (day/cm)	External fixator index (day/cm)	Follow-up period	Paley scale of difficulty	Results of femur	Results of tibia	Functional results of tibia
1	M	Malunion	Shortness	241	50	5	10	48.2	31	4	95 – excellent		
2	F	Congenital	Coxa vara 10°, genu varum 10°, shortness	266	60	6	10	44.3	17	5	90 – good		
3	F	Malunion	Genu varum 10°, shortness	240	20	2	10	120	19	6	95 – excellent		
4	M	Malunion	Genu valgum, shortness	149	30	3	10	49.6	14	3	65 – fair		
5	M	Perthess	Shortness	214	40	4	10	53.5	13	5	75 – good		
6	M	Malunion	Genu varum 10°, shortness	210	57	5	11.4	42	11	4	80 – good		
7	M	Congenital	Genu valgum 14°	104	10	1	10	104	12	1	90 – good		
8	M	Congenital	Shortness	180	35	3.5	10	51.4	11	1	95 – excellent		
9	M	Congenital	Shortness genu valgus 8°	150	20	2	10	75	6	7	90 – good		
10	M	Malunion	Genu varum 20°, procurvatum 10°	210	30	3	10	70	14	3	95 – excellent		
11	F	Malunion	Tibial internal rotation 20°	114	13	1	13	114	24	1		excellent	excellent
12	M	Multiple Enkondromatozis	Tibial procurvatum 30°, valgus 20°	161	10	1	10	161	20	3		good	good
13	M	Congenital	Shortness	219	40	4	10	54.7	19	3		excellent	excellent
14	M	Congenital	Shortness	300	50	5	10	60	15	2		excellent	excellent
15	M	Osteogenesis imperfekta	Tibial procurvatum 10°, varus 10°	265	14	1	14	265	16	6		excellent	good
16	M	Congenital	Tibial procurvatum 5°, varus 14°	210	10	1	10	210	13	5		poor	good
17	M	Malunion	Tibial procurvatum 5°, valgus 15°	213	20	2	10	106.5	7	6		excellent	excellent

Superficial pin site infection was observed in all patients and was successfully treated with oral antibiotics. In one patient, who underwent surgery to treat a femur genu valgus deformity with soft tissue release, a superficial infection developed that did not respond to oral antibiotics. After the Schanz screws were removed under polyclinic conditions, the infection was resolved. Intravenous antibiotherapy was not required for any patient.

In one patient with malrotation (14° in the coronal plane) because of malunion in the left ankle joint, a supramalleolar percutaneous osteotomy was performed with a Gigli saw. During follow-up, union was not observed, so the external fixator was removed. Three weeks later, grafting was performed, and a distal tibia plate was applied.

More than 20° flexion loss was observed in the follow-up of two patients with 4- and 5-cm shortness who underwent femur lengthening with a distal femur osteotomy. This loss was resolved with physical therapy after the fixator was removed. The patient with 4-cm shortness achieved a joint range of motion of 120°, and the patient with 5-cm shortness achieved a joint range of motion of 130°.

After the fixator was removed, one patient who underwent 5-cm isolated lengthening of the tibia fell during follow-up, and angulation was observed in the newly formed callus tissue. The patient had no complaint, and a 5° procurvatum deformity was determined in the lateral plane in the tibia. A second operation was not considered, and the patient was monitored conservatively. In the 7th postoperative month, one patient with 6-cm lengthening of the femur fell while running for exercise, and a fissure was observed in the distraction segment. A brace was applied to the internal femoral area, and the patient was followed up conservatively. No new deformity developed.

## Discussion

External fixators are a basic means of treatment for deformities [6]. When using external fixators, there are certain treatment principles. The smallest mistake at the planning stage leads to large deformities after treatment. To avoid such situations, computer-assisted spatial fixators have come into current use [2].

New mechanical features of these fixators have greatly facilitated multi-axial deformity surgery. If the lengths of the telescopic rods and the diameters of the rings to be used are known, the placement of these rings in relation to each other can be calculated mathematically. Various computer software programs are used to speed up this process and provide ease and convenience to the surgeon [7]. The current study used the Spider Frame Correction Software, high-tech software that allows for the correction of all deformities at the same time.

Because strut changes can be made in the system without surgery, the additional burden to both the patient and physician is minimized. If the hexapod system is assembled correctly, residual deformities can be corrected by changing struts only when necessary, without the need for any other modification. Because corrections can be made at the same time in patients with multi-axial deformities, the distraction time is shorter than that of traditional systems [8]. In the current study, in which the Spider Frame was applied to 17 patients, a strut change was necessary in one case, and this was performed under polyclinic conditions.

When Ilizarov applications are not planned correctly, residual deformities may remain. When the hexapod system is used, residual deformities may develop after correction. To reduce the error rate to a minimum and avoid residual deformity, it is crucial that the reference ring is mounted orthogonally to the bone segment during the operation and that the reference ring is completely parallel to the ground and its position verified by radiograph. In the current study, the first application of the reference ring was placed perpendicular to the bone under fluoroscopy guidance; therefore, no major errors occurred. In previous studies, a radiolucent platform was developed to allow the reference ring to be fixed fully perpendicular to the ground [9]. Thus, the number of radiographs required to obtain the correct image is kept to a minimum. The patients in the current study were positioned by the doctor to ensure that correct postoperative radiographs were taken.

In a study by Marangoz et al. [10], femur deformities were corrected with a Taylor spatial frame, and the mean External Fixator Index was reported to be 66 days/cm. In the current study, the mean External Fixator Index was 67 days/cm (range, 38–300 days/cm). The literature reports variations in mean External Fixator Index values because different cases with different difficulty levels had different durations of external fixator use.

Sakurakichi et al. [11] reported that a lengthening of less than 3 cm extended the External Fixator Index. In a study by Hidenori Matsubara et al. [12] of cases that underwent gradual correction, the Distraction Index and External Fixator Index values were lower than those of cases where acute correction was applied. In the current study, acute correction was not applied, and the mean Distraction Index and External Fixator Index values were consistent with the literature.

In the correction of complex deformities, the surgeon must have a certain level of experience to use the classic Ilizarov method [13]. The process involving a computer-assisted hexapod system is shorter than the classic Ilizarov method [14]. In the current study, when creating repeat protocol prescriptions according to the follow-up radiographs, a mean of four correction prescriptions was required for each of the first five cases, but for the last five cases of the study, which used a hexapod system, a mean of two prescriptions per patient was required. Manner et al. [15] stated that even though the learning curve for the hexapod fixator system is shorter than for the classic Ilizarov method, it is still dependent on the surgeon’s experience with the Ilizarov method.

The functional and bone results of tibia lengthening reported in the literature are generally successful. Bone results of 90–96% and functional results of 92–96% have been reported in the literature as excellent and good, respectively [16, 17]. The tibial lengthening scores of the current study were lower than those reported in the literature. In a study of femoral lengthening by Paley using his own criteria, excellent results of 94% were reported [3]. The mean femoral lengthening score of 84% in the current study was lower than the values reported in the literature [3].

Although computer-assisted fixators have relative advantages compared with classic circular external fixators, their high cost has been reported as a disadvantage in the literature [18]. Because computer-assisted fixators are simple to use, easy to understand, and provide the possibility of correcting residual deformities, the costs could be overlooked.

The most restricting factor of the Ilizarov device is the need to modify the frame and correct deformities simultaneously with lengthening [19, 20]. The Taylor Spatial Frame, which uses the Ilizarov device on a Stewart platform concept, remains faithful to the Ilizarov principles [21]. Using virtual hinges, multi-axial deformities can be corrected at the same time, with no need for any modification to the ring other than strut changes [22].

**Figure 8.**
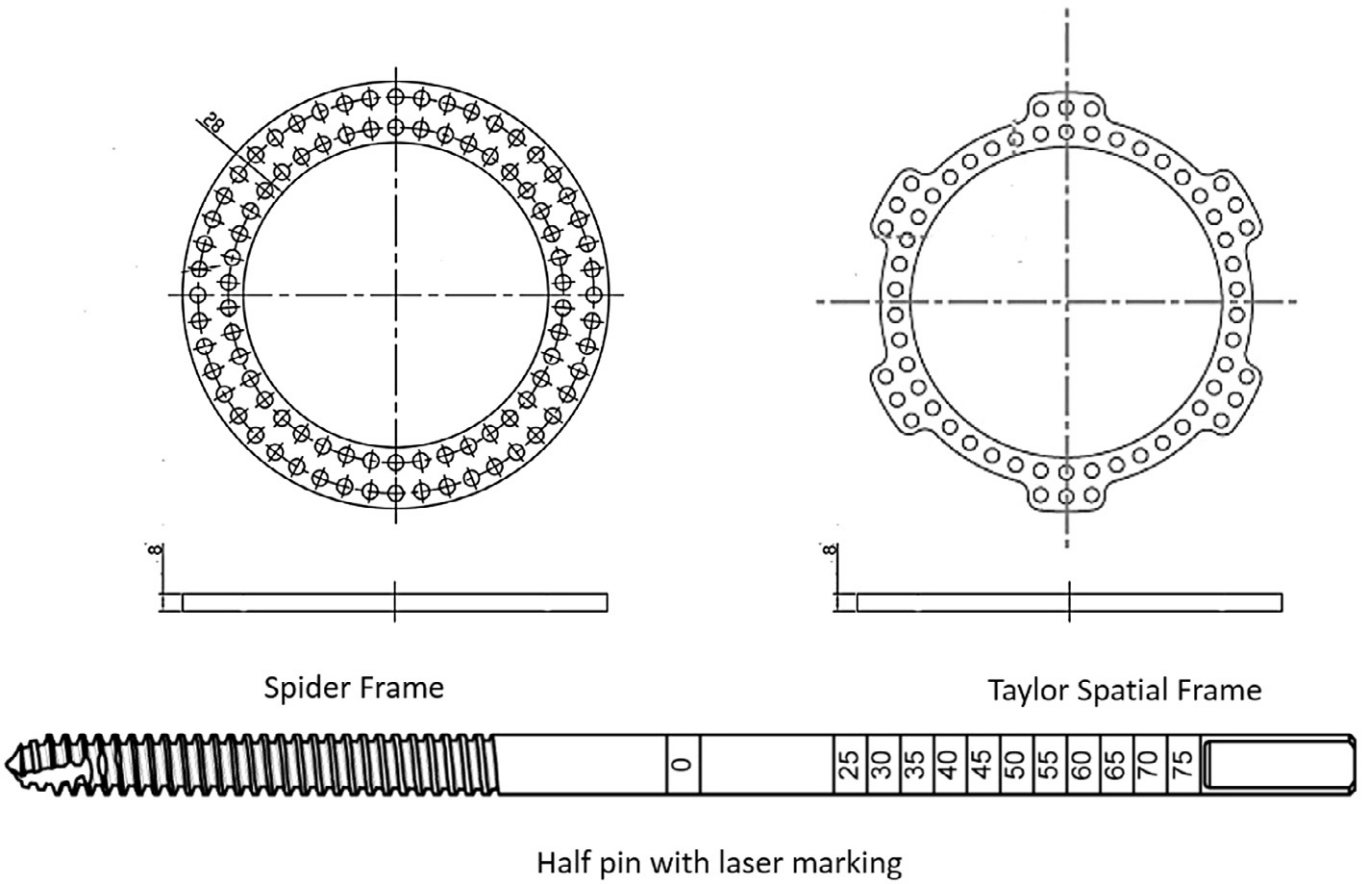
Spider Frame half pins are laser marked with a depth scale, enabling the surgeon to determine the depth to which the half pin is implanted by checking its scale. The rings in the Spider Frame range from 100 mm to 300 mm. Unlike predicate devices, the Spider Frame ring has double-sided holes on its surface.

The Taylor Spatial Frame has been used as a computer-assisted fixator since the 1990s. It is a hexapod frame that consists of two rings or partial rings connected by telescopic struts at special universal joints. When the Taylor Spatial Frame is compared with the classic Illizarov frame, it has the advantage of correcting all deformities simultaneously, which saves time [23]. We used the Spider Frame because it combines the benefits of the Taylor Spatial Frame with some additional advantages. The struts of the Spider Frame are made of titanium alloy. This material has better mechanical properties than the aluminum alloy struts used in the Taylor Spatial Frame. The Spider Frame’s half pins have a laser-engraved depth gauge on their surface (Figure 8). Therefore, the surgeon can insert a half pin more precisely. The Spider Frame software allows the surgeon to select an advanced correction mode. In this mode, the surgeon can choose what to correct first and for how many days. For example, the surgeon can choose to correct AP translation followed by axial translation, lateral translation, and finally angulation. The Spider Frame software has embedded measurement capabilities that can upload an X-ray image and calculate the deformity and reference ring parameters. Unlike predicate devices, the Spider Frame ring has double-sided holes on its surface. This increases the thickness of the Spider Frame rings, allowing them to provide more flexural strength and more connecting holes (Figure 8). The Spider Frame has fast-positioning struts, such as Fast Fx struts. However, the Spider Frame uses a threaded locking mechanism to secure the struts, which is safer for the surgeon because it prevents damage to surgical gloves from the pull and push mechanism. Deformity is always described with respect to the reference bone fragment at the Spider Frame. The Spider Frame has a multi-functional ring that can be used to apply the frame unilaterally.

In cases of both deformity and shortness, the computer-assisted external fixator system (Spider Frame) provides single-stage correction that is easy to use; it does not require system modification, and because of the high-tech software, it has the capability to correct all parameters at the same time, thus allowing postoperative intervention. These features can increase physician confidence and improve the patient’s comfort during the treatment process; therefore, this system is a new generation of external fixator systems that could be preferred because of its many technical advantages.

## Conflict of interest

Authors declare that they have no conflict of interest in relation with this paper.
